# The relationship between sleep quality and menopausal symptoms among postmenopausal women in Saudi Arabia

**DOI:** 10.15537/smj.2022.43.4.20210682

**Published:** 2022-04

**Authors:** Enas M. Abdelaziz, Nadia B. Elsharkawy, Sayeda M. Mohamed

**Affiliations:** *From the Department of Nursing (Abdelaziz, Elsharkawy), College of Applied Medical Sciences, Jouf University, Sakaka, Kingdom of Saudi Arabia; from the Department of Maternal and Newborn Health Nursing (Elsharkawy), and from the Department of Psychiatric Mental Health Nursing (Abdelaziz, Mohamed), Faculty of Nursing, Cairo University, Cairo, Egypt.*

**Keywords:** sleep quality, menopausal symptoms, menopausal rating scale, Pittsburgh sleep quality index, postmenopausal women

## Abstract

**Objectives:**

To assess sleep quality and examine its relationship with menopausal symptoms among Saudi postmenopausal women.

**Methods:**

We carried out a cross-sectional study of 410 postmenopausal women, aged 50-60 years, visiting Prince Mutaib bin Abdulaziz Hospital, Maternity and Children Hospital, and primary health care clinics, Sakaka, Jouf, Saudi Arabia. The menopause rating scale (MRS) was used to assess menopause symptoms and severity, while the Pittsburgh sleep quality index (PSQI) was used to assess sleep quality.

**Results:**

The participants’ age was 53.04±4.15 years, their mean age at natural menopause was 49.14±3.07, and the meantime since their menopause was 6.50±3.84 years. The PSQI total mean score was 6.10±4.17, classified into good versus poor sleepers; 65.4% scored ≤5, and 34.6% scored >5. The Mann-Whitney analysis revealed that somatic and urogenital symptoms, and total MRS score were associated with poor sleep quality (*p*<0.001).

**Conclusion:**

The study findings revealed that more than one-third of Saudi postmenopausal women had poor sleep quality.

Menopause is a natural physiologic event for women in midlife, defined as the permanent cessation of menses for at least one year of amenorrhea after the final menstruation. This occurs due to the aging of the ovaries leading to decrease secretions of estrogen and progesterone and not including factors such as chemotherapy, gland disorders, and hysterectomy.^1^

With increased life expectancy, women can spend more than one-third of their lives in the postmenopausal state.^2,3^ It is estimated that 1.2 billion women will be menopausal by 2030, with 47 million new additions each year globally.^4,5^ Women’s menopause can be influenced by genetic, nutritional, environmental, and psychological factors.^6,7^ During menopause, women may encounter declining physical well-being and climacteric symptoms, including vaginal dryness, hot flashes, sweating, nervousness, stress, mood swings, poor concentration, difficulty with memory, and sleeplessness.^2^ The onset of menopause varies across countries, but the average age is around 50-52 years and most Saudi women reach menopause between the ages of 51-55.^7^

Change in estrogen levels cause irregularities in the menstrual cycle and is considered the first sign of menopause. As estrogen deprivations increase, major somatic and psychological problems originate that influence a woman’s well-being. Early somatic indicators of estrogen deprivation are hot flashes, sweating, headache, and sleep disorders. In contrast, late manifestations are mood swings, cardiovascular disease, osteoporosis, urogenital changes, fatigue, decreased sexual desire, stress incontinence, depressed mood, crying, concentration difficulties, and poor memory.^8^

Sleep plays an essential role in the well-being of an individual. It is a vital physiological process that affects physical, neurological, and psychological functions.^9^ Sleep disorder is the most prevalent and clinically prominent symptom observed during menopause and among the elderly. It is linked to unfavorable health outcomes, such as exhaustion, poor daytime function, and increased visits to healthcare providers.^10^ Numerous researches have sought to find out the causes of sleep disturbances and have discovered that hormonal changes, somatic symptoms, natural aging, and stressful life events can all affect sleep quality.^10,11^ Aging, obesity, hypertension, smoking, and a lack of physical activity have all been associated with sleep disturbances.^12^ The prevalence of sleep disturbances affects 39-47% of perimenopausal women and 35-60% of postmenopausal women. These rates are concerning and may require intervention by health care providers.^13-15^ Poor sleep has various negative consequences, including poor physical, psychological, cognitive, and social outcomes.^15^ Sleep duration strongly influences health, and various studies support the association between sleep problems such as sleeplessness, sleep disturbances, night arousals, excessive daytime sleepiness, apnea, depression, and hot flashes with menopause in women.^13^ Moreover, insufficient sleep has been linked to an increased tendency to gain weight and ultimately, develop diabetes, osteoporosis, and increased fracture risk.^16,17^

Thus, health problems related to the postmenopausal period are essential public health concerns in women at the transitional period of life. Health care providers, such as nurses, dietitians, midwives, and doctors must be sensitive and responsive to a woman’s needs during this stage of life. They should provide guidelines and design health education programs that emphasize adopting healthy and active lifestyles, including weight reduction, physical activity, a healthy diet, maintaining regular medical follow-up to improve the general well-being, and minimize the severity of menopause symptoms. The increased number of postmenopausal women raising concerns regarding their health and sleep. In addition, only few studies have researched the topic in Saudi Arabia and there is limited literature available from the Arab countries. This present study examines the association between sleep quality and menopausal symptoms in Saudi Arabian postmenopausal women.

## Methods

A cross-sectional study of postmenopausal women attending or accompanying patients visiting outpatient clinics at Prince Mutaib bin Abdulaziz Hospital, Maternity and Children Hospital, and primary healthcare clinics, Sakaka, Jouf, Saudi Arabia, between January and April 2021, were selected based on personal interview for the study. The inclusion criteria included all postmenopausal women between 50-60 years old, having menopause for at least one year and voluntarily ready to participate in the study. The exclusion criteria included women who received psychiatric drugs, hormone replacement therapy, undergoing hysterectomy, or having any acute or chronic surgical conditions, cancer, and cognitive impairments, or physical handicap. A sample of 373 was calculated using Roasoft sample size calculator.^18^ The required sample size was computed using a total population size of 12.704 women aged 50-60 years in Sakaka, Jouf, Saudi Arabia, 5% margin of error, 95% confidence interval, and 50% response rate. A large convenience sample of 410 postmenopausal women were recruited to adjust to the dropout rate. The researchers interviewed the eligible participants using a face-to-face structured interview. The study questionnaire was self-administered, and only if the participant could not read or write, the researcher completed the questionnaire based on the participant’s response.

To achieve the study objectives, a 3-structure sectioned and validated questionnaire was used. The questionnaire was administered in the Arabic language to verify that the items were understood by the participants. Before the data collection process, the structure and clarity of this Arabic version were piloted with 40 Saudi postmenopausal women, and no changes to the questionnaire were recommended and the pilot study data were excluded.

Demographic characteristics included information on the participants’ age, education, occupation, marital status, smoking habit, physical exercise, age at menopause onset, time since the menopause onset, having chronic illnesses, and parity were collected. In this section, each participant’s height and weight were measured during the interview to assess their body mass index (BMI) that was calculated by dividing body weight (kg) by height squared (m). Body mass index was classified into 4 groups based on World Health Organization cut-off points: underweight (<18.5), healthy weight (18.5-24.9), overweight (25-29.9), and obese (≥30).^19^

Menopausal rating scale (MRS) is a self-reported standardized Likert scale covering 11 items related to aging symptoms or complaints and was developed by Schneider et al.^20^ Menopausal rating scale was translated from English into simple, understandable Arabic language, which is appropriate for Arab culture by Sweed et al^21^ and was used in the present study. The MRS was categorized into the following 3 subscales: psychological symptoms (4 items that included depressive mood, irritability, anxiety, physical, and mental exhaustion), somatic symptoms (4 items that included sweating/hot flashes, sleep problems, heart discomfort, and joint and muscular discomforts), and urogenital symptoms (3 items that included bladder problems, sexual problems, and dryness of vagina). Each item was scored on a 5-point Likert scale ranging from 0 (no symptoms) to 4 (very severe symptoms). The total score was calculated by adding all the points from each item. The scores ranged from 0 (asymptomatic) to 44, indicating the highest level of complaint and reliability (0.87).^1^ The severity classification summation scores were none (0-4), mild (5-8), moderate (9-16), severe and very severe (≥17). Menopausal rating scale validity and reliability were preserved in the Arabic version; with 90% test-retest agreement.^21^ In this study, Cronbach’s alpha was 0.81 indicating good reliability.

Pittsburgh sleep quality index (PSQI) is an efficient self-reporting scale for measuring subjective sleep quality and sleep patterns, developed by Buysse et al.^22^ An Arabic version is available.^23^ The PSQI distinguishes between “poor” and “good” sleeper by evaluating different aspects of sleep using 7 components and 19 items. Responses were scored on a scale of 0-3. Whereas, 3 represented the adverse extreme of the Likert scale. The researchers added the sum of 7 components to calculate the global PSQI score, which ranged from 0-21; a score of >5 denoted a “poor” sleeper, while a cut-off point of ≤5 indicates a “good” sleeper. Thus, a score of 5-7 indicated the need for medical assessment; 8-14 recommended the need for care and medical treatment, and 14-21 suggested a serious sleeping problem. The Arabic version of the PSQI was tested with 35 Arabic bilinguals, and the documented internal consistency reliability was borderline acceptable (Cronbach’s alpha 0.65).^23^ The scale had good internal reliability in this study (Cronbach’s alpha of 0.83).

The Local Committee of Bioethics at Jouf University, Saudi Arabia approved the study protocol (no.: 03-03-42) in accordance with the Helsinki Declaration principles. The study was described to the director and nurses’ supervisors at the hospital and clinics for approval to carry out the study and facilitate the data collection process. The study’s purpose, design, and benefits were explained to the participants, and written informed consents were collected before they were asked to complete the questionnaire. The participants were informed that the study was voluntary and that they had a right to withdraw anytime. Code numbers were created for each participant, and confidentiality of data was maintained.

### Statistical analysis

Statistical Package for the Social Sciences for Windows, version 20.0 (IBM Corp., Armonk, NY, USA) was used for all statistical analyses. Cronbach’s alpha was used to determine reliability. The Kolmogorov-Smirnov test was used to verify the normality of the distribution. Frequencies and percentages were calculated for categorical variables; means and standard deviations were measured for continuous variables. Mann-Whitney test was used to compare between 2 categories. Kruskal-Wallis test was used to compare between more than 2 categories and post hoc (Dunn’s multiple comparisons test) for pairwise comparisons. The relationship between MRS scores and PSQI scores was explored by Pearson’s correlation coefficient. A *p*-value of <0.05 was considered significant.

## Results

The characteristics of the 410 postmenopausal Saudi women are presented in Table 1. Their mean age was 53.04±4.15 years. Their mean age at natural menopause was 49.14±3.07, and the meantime since their menopause was 6.50±3.84. Most of the participants were married (86.8%) and housewives (74.9%). The BMI was 29.09±5.62 kg/m^2^, and 77.3% were overweight and obese. Aprroximately 70.2% could not read and write and have a primary level of education. The mean number of children was 5.11±1.62. Most participants (60.7%) had chronic illness and 96.8% did not smoke. Hot flashes and sweating were reported by 53.4% and 28.8% of the participants that varied from once to more than 3 times per week. Pairwise comparisons of frequency of hot flashes using Dunn’s post-hoc test indicated that one time per week hot flashes were observed to be significantly higher than not repeated (*p*=0.002). The post-hoc tests also indicated that poor sleep quality was seen more among the younger participants (50-52 years) in the early years of the postmenopausal period than other groups (*p*<0.001), women who had menopause duration of 5-10 years had poor sleep quality than other groups (*p*<0.001). The obese participants (37.1%), had poor sleep quality than normal and overweight (*p*<0.001; Table 1). The mean night sleeping time was 6.20±1.40, indicating short sleep duration, and the total mean scores of PSQI was 6.10±4.17. In categorizing good versus poor sleepers, 268 (65.4%) participants reported good sleep quality and had global scores of ≤5, whereas 142 (34.6%) reported poor sleep quality (PSQI score of >5).

**Table 1:** - Demographic characteristics and factors associated with sleep quality in postmenopausal women (N=410).

Characteristics	Total	Sleep quality			
	n (%)	Mean±SD	Median	Test of sig.	*P*-value
***Age (years), mean±SD***	53.04±3.15				
50-52	202 (49.3)	7.28±4.28	5.04.03.0	H=24.779*	<0.001*
53-55	128 (31.2)	6.97±4.38		
56-60	80 (19.5)	5.08±3.75			
***Time since menopause onset, mean±SD***	6.50±3.84				
<5 years	181 (44.1)	4.85±3.46	3.0		
5-10 years	153 (37.3)	7.86±4.54	10.0	H=27.713*	<0.001*
≥10 years	76 (18.5)	6.71±4.32	5.0		
***Education***					
Cannot read and write/primary	288 (70.2)	5.78±4.02	4.0		
Intermediate/secondary	73 (17.8)	6.25±4.26	4.0	H=1.350	0.509
University/Master - PhD	49 (12.0)	7.43±4.42	10.0		
***BMI, mean±SD***	29.09±5.62				
Underweight	15 (3.7)	5.93±4.22	4.0		
Normal - health weigh	78 (19.0)	4.69±3.23	3.5	H=19.682*	<0.001*
Overweight	165 (40.2)	5.80±4.24	4.0		
Obese	152 (37.1)	7.16±4.29	5.0		
***Parity, mean±SD***	5.11±1.62				
0	9 (2.2)	10.33±4.00	11.0		
1-4	136 (33.2)2	5.86±3.97	4.0	H=9.338*	0.009*
≥5	65 (64.6)	6.57±4.72	4.0
***Marital status***					
Single	9 (2.2)	9.44±2.74	10.0		
Married	356 (86.8)	6.08±4.22	4.0	H=5.785	0.055
Widowed or divorced	45 (11.0)	5.56±3.75	4.0		
***Occupation***					
Housewife	307 (74.9)	5.93±4.07	4.0		
Employee	63 (15.4)	6.67±4.58	4.0	H=1.350	0.509
Retired	40 (9.8)	6.45±4.31	4.5		
***Smoking***					
Yes	13 (3.2)	6.62±4.57	4.0		
No	397 (96.8)	6.08±4.16	4.0	U=2376.5	0.624
***Exercise***					
Yes	98 (23.9)	6.05±4.14	3.0		
No	312 (76.1)	6.24±4.29	4.0	U=490.5	0.316
***Having chronic illnesses***					
Yes	249 (60.7)	6.71±4.32	5.0		
No	161 (39.3)	5.15±3.75	3.0	U=15655.0*	<0.001*
***Frequency of sweating***					
Not been repeated	292 (71.2)	5.96±4.10	4.0		
Once a week	59 (14.4)	5.76±4.02	4.0	H=7.720	0.052
Twice a week	45 (11.0)	6.42±4.09	5.0		
3 times a week or more	14 (3.4)	9.29±5.58	11.0		
***Frequency of hot flashes***					
Not been repeated	191 (46.6)	4.48±3.39	3.0		
Once a week	136 (33.2)	5.90±3.93	4.0	H=83.673*	<0.001*
Twice a week	37 (9.0)	10.27±2.78	11.0		
3 times a week or more	46 (11.2)	10.02±3.97	11.0		

H: for Kruskal-Wallis test, pairwise comparison between each 2 groups was carried out using post hoc test (Dunn’s for multiple comparisons test), U: Mann-Whitney test, statistically significant at *p*<0.05, SD: standard deviation, sig.: significance, BMI: body mass index

Table 2 shows the total MRS and the subscale score according to poor and good sleep quality. Somatic (*p*<0.001), urogenital symptoms (*p*<0.001), and total mean MRS score (*p*<0.001) were associated with poor sleep quality. Several somatic symptoms including hot flashes and sweating, sleep problems, and joint and muscular discomfort, and urogenital symptoms including bladder problems, vaginal dryness, and sexual problems were significantly related to poor quality of sleep. Psychological symptoms were not associated with poor sleep quality (*p*=0.095).

**Table 2:** - Total menopausal rating scale and subscale scores with sleep quality in study participants (N=410).

Menopausal symptoms	Sleep quality				U	*P*-value
	Poor >5 (n=142)		Good ≤5 (n=268)			
	Mean±SD	Median	Mean±SD	Median		
***Somatic symptoms***	4.77±2.87	5.0	3.44±2.56	3.0	13264.0*	<0.001*
Hot flashes, sweating	1.58±1.17	2.0	1.01±1.06	1.0	13666.5*	<0.001*
Heart discomfort	0.66±0.86	0.0	0.56±0.79	0.0	17942.0	0.279
Sleep problems	1.19±1.0	1.0	0.87±0.89	1.0	15666.0*	0.002*
Joint and muscular discomfort	1.34±1.12	1.0	1.0±1.07	1.0	15671.5*	0.002*
***Psychological symptoms***	4.0±3.48	4.0	3.40±3.45	3.0	17108.0	0.084
Depressive mood	0.90±1.01	1.0	0.77±1.01	0.0	17509.0	0.148
Irritability	1.23±1.14	1.0	1.03±1.12	1.0	17190.0	0.087
Anxiety	0.63±1.0	0.0	0.67±0.93	0.0	18156.5	0.383
Physical and mental exhaustion	1.22±1.10	0.0	0.94±1.07	0.0	16190.0*	0.009*
***Urogenital score***	4.88±2.37	5.0	3.93±2.64	4.0	14943.5*	<0.001*
Bladder problems	1.87±0.89	2.0	1.60±1.04	1.0	15921.0*	0.004*
Vaginal dryness	1.53±1.0	2.0	1.29±1.23	1.0	16649.5*	0.031*
Sexual problems	1.49±1.14	1.0	1.04±1.14	1.0	14756.0*	<0.001*
Total	13.75±6.77	13.0	10.71±6.52	9.0	13379.5*	<0.001*

Statistically significant at *p*<0.05, U: Mann-Whitney test, SD: standard deviation

Pearson correlation coefficient revealed significant weak correlations with the total PSQI score, including the total MRS score (r=0.210), the somatic symptoms (r=0.228), and a very weak correlation with urogenital symptoms (r=0.177), suggesting the worse menopausal symptoms, the poor sleep quality (*p*<0.001; Table 3).

**Table 3:** - Pearson correlation coefficient between subscales of menopause rating scale and sleep quality.

Menopausal symptoms	Total sleep	
	**r**	*P*-value
Somatic symptoms	0.228*	<0.001*
Psychological symptoms	0.082	0.098
Urogenital symptoms	0.177*	<0.001*
MRS total scale	0.210*	<0.001*

Statistically significant at *p*<0.05, r: Pearson coefficient, MRS: menopause rating scale

## Discussion

The study results showed that most participants (65.4%) had good sleep quality, which may be related to the good living condition, high economic status, and high quality of medical services in Saudi Arabia. However, approximately one-third (34.6%) had poor sleep quality that necessitated medical attention. This finding was consistent with Kim et al^24^ who reported that 30.2% of South Korean postmenopausal women had poor sleep quality. A Canadian longitudinal study by Zolfaghari et al^25^ showed that 32.4% of women expressed poor sleep satisfaction, and Creasy et al^26^ revealed that 35% of postmenopausal women had short sleep duration of ≤6 hours per night in the United States. While Valencia et al^13^ found that nearly half (46.7%) of Argentinian women had poor sleep quality. Middle-aged Chinese women had experienced sleep disturbances with a total PSQI score of 8.58±4.37.^27^ In an Iranian study, 56.3% of postmenopausal women were identified as poor sleepers.^28^ A naturally postmenopausal women among Shanghai residents, China, had poor sleep quality by 12.5%.^29^ The variations in the results could be attributed to biological, psychosocial, socioeconomic, cultural, and racial/ethnic factors. Moreover, women are more likely to have disturbances of sleep due to estrogen declining during menopause, making them more sensitive to negative emotional information.^30^ Poor sleep quality may increase the risk of cardiovascular diseases, hypertension, obesity, diabetes mellitus, increase healthcare usage, depression, distress, and low quality of life.^13^

This study results were in the same alignment with previous studies indicating a significant association between poor sleep quality and sociodemographic variables (*p*<0.001). Creasy et al^26^ and Blümel et al^32^ stated that an inactive lifestyle had been linked with insomnia in postmenopausal women. Wu et al^29^ showed that chronic disease was linked with sleep disturbance in the middle-aged and elderly. Zhang et al^33^ found that the symptoms of menopause were more prominent during early years of postmenopause.

The study findings revealed a statistically significant association between somatic and urogenital symptoms with poor sleep quality (*p*<0.001). Previous studies have supported these findings which draw a correlation between menopausal symptoms and sleep disturbances.^11,12,24,31,32^ It has been proposed that menopause may have no negative impact on sleep quality and there were other causes for poor sleep quality among postmenopausal such as vasomotor symptoms, estrogen reduction, and the process of aging.^34^ Women with a low level of education or who were uneducated were more susceptible to experiencing poor quality of sleep. Therefore, the present study suggests that a higher educational level positively influences sleep quality. Educated women are less complaining and are more worried on their physical well-being. They intend to seek answers to their health problems, whether through serious research or with the assistance of specialists, and they more often have easier access to healthcare strategies. This finding was consistent with Kim et al^24^ who reported that with increased education level in middle-aged women, the sleep difficulties decreased. Furthermore, there was a link between poor sleep quality and BMI as 40.2% of all participants were overweight, and 49.3% with poor sleep quality were obese with a mean BMI of 30.4±5.87 kg/m^2^. In postmenopausal women, sleep disturbances are caused by higher BMI and abdominal obesity while increasing obstructive sleep apnea.^3,24,32^ Conversely, Zagalaz et al^12^ disagreed with the link between high BMI and poor sleep quality.

### Study limitations

The use of a convenience sample and the inability to conclude cause and effect due to the nature of the cross-sectional research design. In addition, we could not rule out the possibility of other intervening effects of the aging influencing the quality of sleep. Furthermore, the study focused on women from a specific geographic location, it cannot be generalized to the whole Saudi middle-aged women since they do not share the same characteristics as the sample population. Self-reported questionnaires were also used to assess sleep quality and menopausal symptoms, implying the requirement for an objective approach like polysomnography.

In light of this, there is a need to develop effective management strategies to reduce menopausal symptoms and other related factors that may improve sleep quality. There should be more awareness on the importance of education and having a healthy lifestyle. Further interventional studies need to be carried out to establish effective measures to improve sleep quality. The effect of obesity and physical exercise on sleep quality and menopausal symptoms among Saudi women must be examined. A longitudinal study is crucial to assess menopausal symptoms effect on sleep quality among Saudi women, and large-scale national clinical studies are recommended in the future.

In conclusion, more than one-third of Saudi postmenopausal women had poor sleep quality, which needs medical attention. Poor sleep quality seems to be related to somatic and urogenital symptoms. In addition, factors such as uneducated or lower-educated women, obesity, and no physical exercise influence sleep quality among Saudi postmenopausal women.
